# Mindful parenting and preschoolers’ screen dependency behavior: the mediating role of parent–child relationship and the moderating role of effortful control

**DOI:** 10.3389/fpsyg.2026.1785133

**Published:** 2026-06-17

**Authors:** Fangli Liu, Yanqing Yan, Chunrong Hu, Qiuxian Ye

**Affiliations:** 1 Department of Education and Psychological Sciences, Yuncheng University, Yuncheng, China; 2 Sports Department, Guangdong University of Science and Technology, Dongguan, China; 3 Department of Physical Education, Hengshui University, Hengshui, China; 4 Zhuhai College of Science and Techology, Zhuhai, China

**Keywords:** effortful control, mindful parenting, parent–child relationship, PLS-SEM, preschoolers’ screen dependency behavior

## Abstract

**Background:**

In the digital age, screens have become an indispensable part of children’s lives. The phenomenon of preschoolers excessively using screens, even developing screen dependency behavior, has emerged. Mindful parenting is increasingly considered a factor potentially associated with lower levels of children’s screen dependency behavior. However, little is known about the underlying mechanisms and effective interventions, especially under the influence of parent–child relationship and preschoolers’ effortful control.

**Methods:**

Using a stratified cluster sampling method, a questionnaire survey was conducted among parents of preschool children in Shanxi and Hebei provinces of China. We collected a total of 654 valid questionnaires using a combination of offline and online approaches. Using validated instruments, we assessed mindful parenting, preschoolers’ screen dependency behavior, parent–child relationship, and preschoolers’ effortful control. We employed a partial least squares structural equation model (PLS-SEM) to perform data analysis.

**Results:**

Mindful parenting showed a significant direct negative association with preschoolers’ screen dependency behavior (*β* = −0.15, *p* < 0.01), and this association was partially mediated by parent–child relationship (indirect effect *β* = −0.03, *p* < 0.05). Preschoolers’ effortful control significantly negatively moderated the association between mindful parenting and parent–child relationship (*β* = −0.13, *p* < 0.01), and positively moderated the association between mindful parenting and preschoolers’ screen dependency behavior (*β* = 0.09, *p* < 0.01). The model accounted for 49% of the variance in parent–child relationship and 69% in screen dependency behavior.

**Conclusion:**

Findings indicate the importance of integrating parental factors, parent–child relationship, and children’s characteristics in understanding screen dependency behavior in preschool children. This study not only responds to the call for adaptive parenting strategies in the digital age, but also supports the localization and contextualization of temperament-environment interaction theory and attachment theory. It provides a theoretical foundation for subsequent research on individualized parenting adaptation and offers practical suggestions for developing family intervention programs.

## 1 Introduction

In the digital age, screens have become an increasingly indispensable part of children’s lives. Children are exposed to a large amount of screen time from an early age (Abdul Hadi et al., 2022). Premature screen exposure and excessive screen time have become very common among children (Xiang et al., 2022). A study on preschoolers in China showed that about 62% of children use screens for more than 1 h per day, and 60% of children begin to use electronic screens before the age of two (Chen et al., 2021). Children under six experience a critical period of brain development (Bavelier and Neville, 2002). Prior studies have shown that excessive screen exposure during early childhood correlates strongly with cognitive developmental delays, emotional regulation difficulties, and frequent behavioral problems (Radesky et al., 2015; Zhao et al., 2022; Lakicevic et al., 2025). Excessive screen use is also associated with screen dependency behavior among children (Bai et al., 2025). Screen dependency behavior refers to problematic interactions with screen media, including smartphones, tablets, computers, televisions, and portable video games (Hutton et al., 2020), characterized by exhibiting uncontrollable behaviors and negative emotions such as impatience and anxiety in the absence of screens (Abdul Hadi et al., 2022). This behavior can hinder the healthy physical and mental development of children. Therefore, studying the mechanisms of preschoolers’ screen dependency behavior formation and protective variables has become an important topic in developmental psychology and family studies.

According to family systems theory, parenting styles are associated with children’s behaviors (Grusec, 2011; Fu et al., 2024). Mindful parenting, defined as a parenting style grounded in non-judgmental awareness, primarily stresses that parents maintain full attentiveness to their parenting roles and remain focused on the present moment during interactions with their children (Duncan et al., 2009). Previous studies have found that mindful parenting is related to parent–child relationship (Huang et al., 2025), children’s creative tendencies (He et al., 2024), emotional regulation abilities (Sansone, 2024), and adolescent behavioral adaptation (Acet et al., 2024). A study involving 482 Chinese preschoolers’ parents found that mindful parenting is associated with the extent of excessive screen time use in young children (Zhang, 2025). However, the underlying mechanisms of how mindful parenting is related to preschoolers’ screen dependency behavior remain unclear and require further exploration.

Attachment theory posits that secure attachment is associated with consistent and sensitive interactions between parents and children, and this interaction pattern correlates with the quality of parent–child relationship. A positive and warm parent–child relationship correlates with the fulfillment of children’s psychological needs and a weaker tendency to seek comfort through external stimuli such as smartphones (Qiu et al., 2022). Mindful parenting, in turn, is linked to the formation of a high-quality parent–child relationship. By enhancing parents’ awareness during the parenting process, promoting their emotional regulation abilities, and increasing their sensitivity to their children’s responses, mindful parenting is associated with improved quality of parent–child activities and is linked to positive parent–child relationship (Huynh et al., 2024). However, the mediating mechanism of parent–child relationship between mindful parenting and preschoolers’ screen dependency behavior still needs further exploration.

Children’s behavioral patterns and social–emotional development are associated with parent–child relationship and varies in relation to individual factors, such as the individual’s effortful control ability (Coe et al., 2024). Effortful control is the ability of individuals to actively regulate attention, emotions, and behaviors through executive functions such as inhibitory control and attention regulation. It is one of the core dimensions of temperament and an important manifestation of self-regulation at the temperamental level (Rothbart et al., 2000). Existing research has found that an individual’s level of effortful control is significantly associated with their academic performance and is closely related to aggression and antisocial behaviors (Smith and Day, 2018; Sánchez-Pérez et al., 2018). As a consistent and robust predictor of important developmental outcomes in individual behaviors (Moffitt et al., 2011), effortful control has been shown to be related to the mitigation of adverse effects associated with negative parenting or caregiving difficulties in children (Coe et al., 2024). Therefore, it can be inferred that during the formation of screen dependency behavior, children’s effortful control may constitute an internal protective barrier, mitigating the impact of adverse environments. However, the moderating mechanism through which effortful control operates between mindful parenting and preschoolers’ screen dependency behavior remains to be further explored.

Based on this background, while prior research has individually examined the associations of mindful parenting, parent–child relationships, and effortful control with children’s behavior, it remains unclear how these three factors are jointly related to screen dependency behavior in preschool children. This study aims to examine the relationships among mindful parenting, parent–child relationship, effortful control, and screen dependency behavior in preschool children, with a focus on analyzing the mediating role of parent–child relationship and the moderating role of effortful control. It is important to note that, due to the cross-sectional design, the proposed mediation model does not aim to establish temporal causality. Instead, it is theoretically derived from family systems theory, attachment theory, and the temperament–environment interaction perspective. Mindful parenting is theoretically linked to parent–child relationship, and this relationship is further theorized to correspond to screen dependency behavior. Thus, the indirect association is examined as a theoretically plausible pathway, not as evidence of causal ordering. Preschool children are in a critical period of self-regulation and social–emotional development. Understanding the interaction between family and individual factors may help identify protective factors against screen dependency, providing a basis for promoting healthy media usage habits and enhancing psychological adaptability in young children. This study applies mindful parenting theory to the context of digital media use, thereby providing a new perspective on understanding the developmental mechanisms of children’s screen dependency behavior. The research results may provide empirical support for parents and educators to design more effective parenting programs and screen usage guidelines.

## 2 Literature review and research hypotheses

### 2.1 Mindful parenting and screen dependency behavior

The family environment, especially parenting styles, is significantly associated with the formation of early behavioral patterns in children. Parents’ attitudes toward parenting are related to children’s social–emotional abilities and behavioral issues (Rubin et al., 2002). In recent years, mindful parenting has received great attention as a positive parenting style (Meppelink et al., 2016). Mindful parenting emphasizes that parents respond to their children’s needs in a conscious, non-judgmental, and emotionally balanced manner during parent–child interactions (Duncan et al., 2009), which provides a novel perspective for understanding and managing children’s screen use. Prior studies indicate that higher levels of mindful parenting correlate with fewer internalizing and externalizing problems among children (Vernon and Moretti, 2024). A 10-month longitudinal study involving parents of elementary school children from two schools in Guangdong Province, China, revealed that mindful parenting significantly negatively predicts children’s problematic behaviors (Fu et al., 2024). Accordingly, we propose:

*Hypothesis 1 (H1)*: Mindful parenting is significantly negatively associated with preschoolers’ screen dependency behavior.

### 2.2 The mediating role of parent–child relationship

The parent–child relationship is the earliest social connection formed by individuals. Among the many influencing factors, mindful parenting is positively associated with the establishment of a good parent–child relationship (Kakhki et al., 2022). Prior studies indicate that parents who practice mindful parenting report lower parenting stress, more positive parenting practices, and a healthy parent–child relationship (Potharst et al., 2021). A longitudinal randomized controlled study showed that changes in the level of mindful parenting in both parents are significantly positively correlated with changes in the quality of parent–child relationship (Coatsworth et al., 2018). Mindful parenting has been associated with parents’ ability to regulate negative emotions during the educational process, enabling them to listen to and understand their children’s needs more patiently, which is related to better parent–child relationship quality (Singh et al., 2010). Therefore, we propose:

*Hypothesis 2 (H2)*: Mindful parenting is positively associated with parent–child relationship.

The problem behavior theory posits that dysfunctional family relationships, especially parent–child relationships, are significantly associated with the emergence and exacerbation of adolescent internet addiction (De Leo and Wulfert, 2013). A study shows that parent–child relationship correlates negatively with adolescents’ deviant internet behaviors, and that this relationship may be closely associated with adolescent internet addiction (Kong and Lim, 2012). A meta-analysis indicates that problematic internet use among children and adolescents aged 6–25 is negatively correlated with parent–child relationship (Zhu et al., 2022). A positive and good parent–child relationship is associated with fewer problem behaviors in children (Dykstra et al., 2020). Children in poor parent–child relationships are more likely to exhibit problem behaviors, including rule-breaking and violent behaviors (Xerxa et al., 2020). Therefore, we propose:

*Hypothesis 3 (H3)*: Parent–child relationship is negatively associated with preschoolers’ screen dependency behavior.

According to empirical studies, mindful parenting correlates significantly with children’s decision-making processes, and parent–child relationship is also linked to this association (Wong et al., 2019). A cross-lagged panel study indicates that mindful parenting significantly affects children’s internalizing behaviors by influencing parent–child relationship (Huang et al., 2025). Among parents who practice mindful parenting, higher quality of parent–child communication and more positive parent–child relationships are observed. These two factors, in turn, correlate with children’s behavioral issues (Yang et al., 2022). According to related research, parent–child relationship is a crucial link in the connection between harsh parenting and children’s screen time. Moreover, parenting styles show both direct and indirect correlations with children’s screen time, with parent–child relationship quality as a pathway (Zhang, 2025). The parent–child relationship clearly serves as an important intermediate factor in relation to children’s problematic smartphone use (Shao et al., 2024). Accordingly, we propose:

*Hypothesis 4 (H4)*: Parent–child relationship mediates the relationship between mindful parenting and screen dependency behavior in preschoolers.

### 2.3 The moderating role of effortful control

Parenting styles and children’s effortful control have always been important topics in child development research. Parenting styles are associated with children’s psychological development, personality formation, and behavioral adaptation (Hong et al., 2025). Some studies have found that children’s temperamental characteristics can, to some extent, moderate the relationship between parenting styles and their psychological and behavioral outcomes (Del Puerto-Golzarri et al., 2022). Children with higher effortful control are stronger in self-regulation of behaviors and emotions (Eisenberg et al., 2015). Therefore, effortful control may act as a moderating variable, influencing the relationship between parenting styles and parent–child relationship. As children’s effortful control abilities improve, the positive association between mindful parenting and parent–child relationship may gradually weaken. Conversely, the lower the effortful control, the stronger the positive association between mindful parenting and parent–child relationship. Accordingly, we propose:

*Hypothesis 5 (H5)*: Effortful control negatively moderates the association between mindful parenting and parent–child relationship, such that this association is weaker for preschoolers with higher effortful control.

Previous studies have shown that effortful control plays a crucial role in the early development of children’s externalizing problems (Choe et al., 2013). As an important protective factor, effortful control is associated with the impact of external stressors (Gartstein and Fagot, 2003), and is also related to lower levels of internalizing and externalizing problems through the regulation of negative emotions and cognitive abilities (Van Prooijen et al., 2018). Furthermore, empirical studies have shown that effortful control functions as a significant moderator in the relationship between parenting styles and children’s behavioral problems (Li et al., 2020). The link between parental conflict and children’s externalizing problems is significant only in the low effortful control group, whereas high effortful control is associated with protection from such adverse effects (Thompson et al., 2020). Therefore, effortful control may moderate the extent to which parenting styles are related to externalizing behaviors in young children, such as screen dependency behavior. Children with lower effortful control are more strongly associated with parenting styles, and mindful parenting shows a stronger association with their screen dependency behavior. Children with higher effortful control have stronger self-regulation ability, and the association between mindful parenting and their screen dependency behavior is less pronounced. Accordingly, we propose:

*Hypothesis 6 (H6)*: Effortful control positively moderates the association between mindful parenting and preschoolers’ screen dependency behavior, such that this association is weaker for preschoolers with higher effortful control.

The differential susceptibility model posits that children’s temperament is associated with environmental influences (Belsky, 1997). Lower effortful control may be associated with stronger harmful associations related to negative parenting, while higher effortful control may be associated with protection from such negative associations (Lengua et al., 2008). A study involving 241 American children at risk of behavioral problems shows that the association between punitive discipline and children’s outcomes varies according to their level of effortful control. Additionally, parenting styles and children’s effortful control interact with their externalizing behaviors (Lee et al., 2024). Previous studies have confirmed that effortful control significantly moderates the association between parent–child relationship and behavioral problems in young children (Coe et al., 2024), providing empirical support for moderated mediation mechanisms. It can be inferred that the mediated association is stronger for children with lower effortful control, while it is weaker for children with higher effortful control. Accordingly, we propose:

*Hypothesis 7 (H7)*: Effortful control negatively moderates the indirect association between mindful parenting and screen dependency behavior through parent–child relationship, such that the mediated association is weaker for children with higher effortful control.

**Figure 1:**
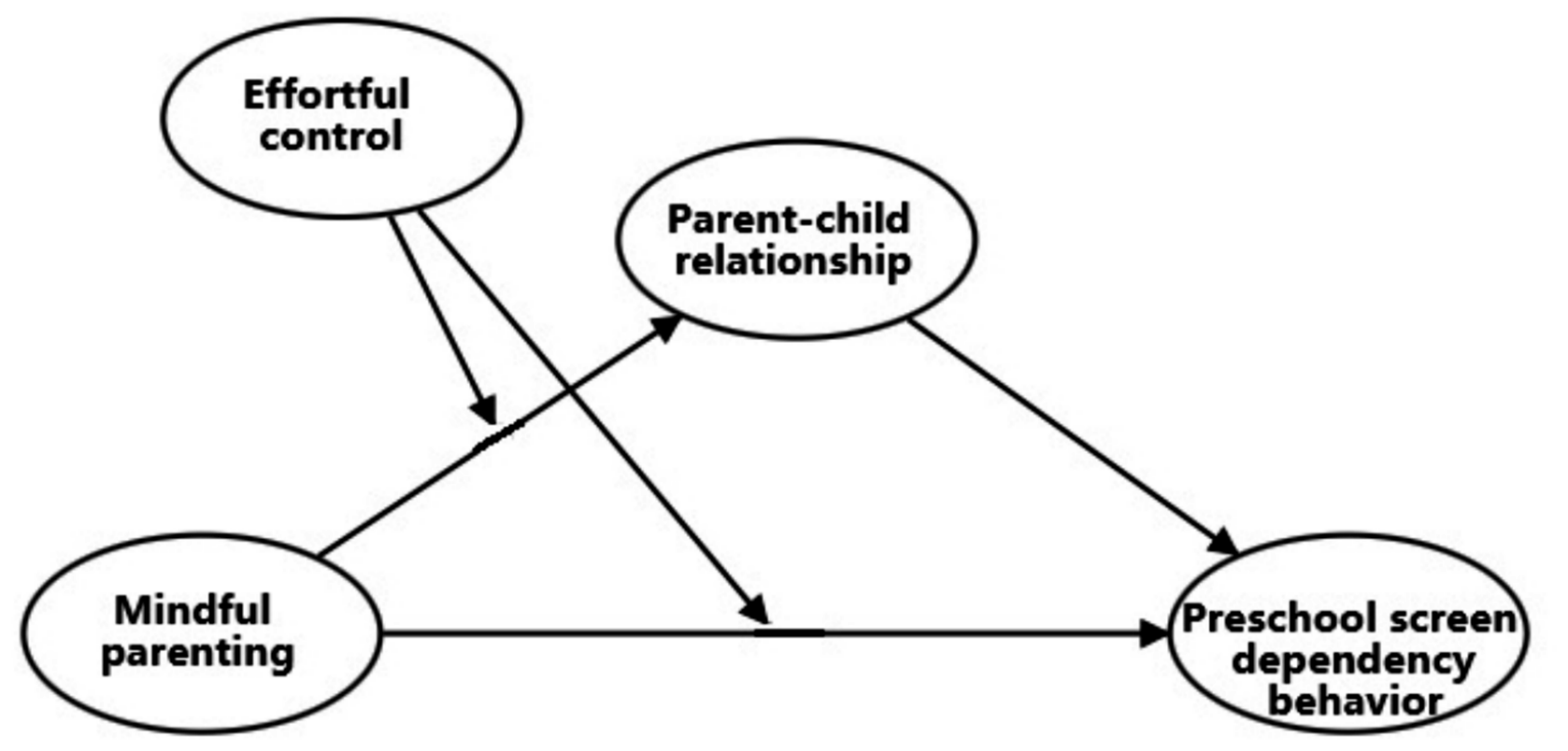
Theoretical model of the relationship between mindful parenting and preschool screen dependency behavior: the mediating role of parent–child relationship and the moderating role of effortful control.

In summary, we have constructed a model primarily aimed at exploring the associations between mindful parenting and screen dependency behavior in preschool children, as well as the underlying mechanisms (see Figure 1). This model specifically investigates the following questions:

First, it examines the direct association between mindful parenting and screen dependency behavior in preschoolers.

Second, it tests whether parent–child relationship mediates the association between mindful parenting and screen dependency behavior in preschoolers.

Third, it examines whether effortful control moderates the direct association and the mediated path between mindful parenting and preschoolers’ screen dependency behavior.

## 3 Methods

### 3.1 Participants

Research participants were recruited from 6 kindergartens, 4 of which were located in Shanxi Province and 2 in Hebei Province, covering a total of 30 classes. A stratified cluster sampling method was adopted based on province and kindergarten type (public vs. private). In Shanxi, 2 public kindergartens each contributed 2 classes from each grade (junior, middle, senior), and 2 private kindergartens each contributed 1 class from each grade, resulting in 18 classes. In Hebei, 1 public and 1 private kindergarten each contributed 2 classes from each grade, resulting in 12 classes. To facilitate participation, the questionnaire was available in both paper and online formats. In Shanxi, we distributed pap questionnaires offline. First, we obtained the principals’ consent, and then we trained the teachers of the surveyed classes, explaining the purpose, process, and precautions of the survey. Next, when parents came to pick up their children from the kindergarten, we distributed paper questionnaires. We collaborated with the teachers to explain the research objectives to the parents in detail and obtained informed consent from all participants. The questionnaire was completed by parents at home and returned to the kindergarten the next day. In Hebei, we distributed electronic questionnaires through the Questionnaire Star platform. After obtaining the voluntary informed consent of the parents, we distributed the electronic questionnaire to the kindergarten teachers, who then forwarded it to the parents.

The paper questionnaire data were independently entered into the database by two professionally trained researchers, and cross-checking was used to reduce entry errors. The electronic questionnaire data were automatically exported by the Questionnaire Star platform. The data collection work lasted for about a month, with a total of 712 questionnaires collected (459 offline and 253 online). After excluding questionnaires with incomplete responses (a total of 77 items with ≥8 missing responses, or complete absence of any entire key variable scale), patterned answers (e.g., selecting the same option for all items, or repetitive cycles such as 1,2,1,2), and online questionnaires completed in less than 4 min, a total of 58 invalid questionnaires were removed. Ultimately, 654 valid questionnaires were obtained (438 offline and 216 online), with an overall validity response rate of 91.85% (95.42% offline and 85.38% online). Among the valid sample, 85.17% of the surveyed parents were preschoolers’ mothers. 53.98% of preschoolers were boys and 46.02% were girls. 31.50% were in the junior preschool class, 29.82% were in the middle preschool class, and 38.68% were in the senior preschool class. 72.48% of parents were aged between 31 and 40. 77.21% of the parents had a college degree or above.

In addition, to test the equivalence of online and offline data collection methods, we used AMOS 28 software and performed multi-group confirmatory factor analysis (MGCFA) to examine measurement invariance. The results showed that the configural invariance model fit well (*χ*^2^ = 4028.251, df = 3,066, CFI = 0.961, TLI = 0.960, RMSEA = 0.022), laying the foundation for subsequent nested model comparisons. After sequentially constraining the factor loadings, intercepts, and error variances, the ΔCFI and ΔTLI of each hierarchical model compared to the preceding model were both less than 0.01, and ΔRMSEA was less than 0.015, meeting the criteria for determining measurement invariance (Cheung and Rensvold, 2002; Chen, 2007). In summary, the measurement scales in this study were equivalent in terms of configural invariance, weak invariance, strong invariance, and strict invariance under both data collection methods. This indicates that the online and offline questionnaires measure the same construct equivalently, without measurement bias due to differences in data collection methods, supporting the subsequent merging of data for overall analysis.

Before conducting the research, we obtained approval from the ethics review board of the first author’s institution (Project No.: KL202506) and secured administrative permission from each participating kindergarten. To protect the rights and interests of participants, the parents were informed before taking part in the study that all data would be handled anonymously and confidentially, and the survey results would only be used for research purposes.

### 3.2 Measures

#### 3.2.1 Mindful parenting

Mindful parenting was measured by the Mindfulness in Parenting Questionnaire (MIPQ), which was designed by McCaffrey et al. (2017) and then translated into Chinese and revised by Wu et al. (2019). The questionnaire has 28 items, divided into two dimensions: “Being in the Moment with the Child” (e.g., “When I talk with my child, I listen attentively and respond to the child.”) and “Mindful Discipline” (e.g., “Before disciplining my child, I consider the child’s feelings.”). The questionnaire is scored from 1 (completely untrue) to 5 (completely true), with elevated total scores signifying a higher level of mindful parenting.

#### 3.2.2 Preschool screen dependency behavior

Preschool screen dependency behavior was measured by the Chinese Preschool Screen Dependency Behavior Scale (CPSD), which was designed by Bai et al. (2025). It is the first standardized screen dependency measurement tool for children aged 3–6 in China. The scale is based on preschoolers’ behavioral characteristics and adopts a three-dimensional structure comprising nine items. (1) Resistance to negative emotions (3 items), which measures children’s negative emotional reactions when access to electronic devices is denied (e.g., “When I take away my child’s mobile phone/tablet, my child becomes upset.”); (2) Physical function impairment (3 items), which assesses the impact of screen use on children’s physical health (e.g., “My child lacks exercise because of using electronic devices.”); (3) Difficulty disengaging from use (3 items), which reflects children’s behavioral control difficulties when trying to stop screen use (e.g., “It is becoming more and more difficult to keep my child away from electronic devices.”). The scale uses a scoring standard of 1 point (almost never) to 5 points (always), with higher totals indicating more serious screen dependency behavior.

#### 3.2.3 Parent–child relationship

The short version of the Child–Parent Relationship Scale (CPRS), originally developed by Pianta (1992), was used to assess parent–child relationship. Zhang and Chen (2010) validated this scale within the Chinese cultural context, and it demonstrates satisfactory reliability and validity. The scale contains two dimensions of closeness and conflict, with a total of 15 items. The closeness dimension measures the open communication, warmth and emotional connection between parents and children, comprising seven items (e.g., “My child can actively share information about himself/herself.”). The conflict dimension assesses the negative interaction or confrontation between parents and children, comprising eight items (e.g., “My child is easily angry with me.”). The scale ranges from 1 (entirely false) to 5 (entirely true). The conflict dimension uses a reverse scoring method, which is then added to the closeness dimension score to yield the overall score of parent–child relationship. A higher score corresponds to a better parent–child relationship.

#### 3.2.4 Effortful control

Effortful control was measured by the simplified version of the Children’s Behavior Questionnaire (CBQ), which was developed by Putnam and Rothbart (2006). The questionnaire comprises three dimensions, of which only the effortful control dimension was utilized in our study. The effortful control dimension subscale contains 12 items, such as “When drawing or coloring in a book, my child shows strong concentration.” and “My child slowly and cautiously approaches places that they are told are dangerous.” The scale is scored from 1 (almost never) to 5 (always), where higher scores correspond to a higher level of effortful control in preschool children.

#### 3.2.5 Control variables

Parenting stress was used as a control variable and measured with the Chinese version of the Parental Stress Scale (Cheung, 2000). The full scale contains 18 items. To keep the measure brief for use as a control variable, we selected seven items with factor loadings > 0.70 based on Cheung’s (2000) principal component analysis. It measures the level of stress parents feel due to their parenting responsibilities (e.g., “I feel overwhelmed by my parenting responsibilities.”). Parenting stress refers to the experience of distress or discomfort arising from the demands associated with the parental role (Abidin, 1992). Previous studies have found that parenting stress is related to family environment, children’s social–emotional and behavioral problems, social support, and parents’ education level (Theule et al., 2018; Fang et al., 2024), and may also affect parent–child relationship (Eo and Kim, 2018). The scale ranges from 1 (completely disagree) to 5 (completely agree). Higher scores are associated with greater parenting pressure among parents.

In addition, we controlled for other variables that may affect preschoolers’ screen dependency behavior, including preschoolers’ age, preschoolers’ gender, highest parental education level, and parents’ screen time.

### 3.3 Data analysis tools

SmartPLS 4.1 software was employed to perform Partial Least Squares Structural Equation Modeling (PLS-SEM) on the data. The selection of this method rested mainly on the considerations below. First, this study involves multiple mediation and moderation paths, such as the mediation of mindful parenting → parent–child relationship → screen dependency behavior, and the moderating role of effortful control on three paths. PLS-SEM can efficiently estimate direct effects, indirect effects, and moderating effects simultaneously, avoiding the multi-step biases of traditional methods (Becker et al., 2012). Second, a sample size of 654 was obtained from the questionnaire in this study, with potential non-normality in the distribution of some latent variable data. However, PLS-SEM has relatively lenient assumptions regarding data distribution, which can ensure the robustness of parameter estimation (Hair et al., 2021). Third, this study investigates the interactive mechanism linking parent–child relationship and effortful control. Compared to the covariance-based structural equation modeling (CB-SEM), which primarily focuses on model validation, PLS-SEM aligns better with the goal of theoretical construction (Urbach and Ahlemann, 2010). Accordingly, PLS-SEM was selected as a suitable method for testing the theoretical framework of this study.

## 4 Results

### 4.1 Evaluation of the measurement model

Table 1 shows that most indicators had factor loadings ranging from 0.70 to 0.90. However, eight items (CPRS2, CPRS5, CPRS15, MIPQ1, MIPQ4, MIPQ5, MIPQ15, and PSS1) had loadings below 0.70. To improve indicator reliability and average variance extracted (AVE), these eight low-loading items were removed from the measurement model. Importantly, this decision was not based solely on statistical considerations. After identifying the low-loading indicators, we re-examined the dimensional coverage of each construct to ensure that the retained items continued to represent the intended conceptual domain.

**Table 1:** Model evaluation.

Constructs	Loadings	VIF	CR	Alph	AVE
Parent–child relationship	0.71–0.80	1.74–2.36	0.93	0.93	0.56
Preschool screen dependency behavior	0.78–0.85	2.13–3.00	0.94	0.94	0.69
Effortful control	0.72–0.80	1.89–2.43	0.94	0.94	0.59
Mindful parenting	0.70–0.78	1.91–2.65	0.96	0.96	0.53
Parenting stress	0.70–0.90	1.98–5.30	0.92	0.91	0.69

Fornell–Larcker	1	2	3	4	5
Parent–child relationship	0.75				
Preschool screen dependency behavior	−0.60	0.83			
Effortful control	0.62	−0.68	0.77		
Mindful parenting	0.67	−0.70	0.75	0.73	
Parenting STRESS	−0.56	0.78	−0.63	−0.65	0.83

HTMT	1	2	3	4	5
Parent–child relationship					
Preschool screen dependency behavior	0.64				
Effortful control	0.67	0.72			
Mindful parenting	0.71	0.73	0.79		
Parenting stress	0.60	0.83	0.67	0.69	

Constructs	*R* ^2^	SRMR	*χ* ^2^	NFI	
Parent–child relationship	0.49	0.04	3205.98	0.89	
Preschool screen dependency behavior	0.69				

Constructs	*f* ^2^	
	Parent–child relationship	Preschool screen dependency behavior
Parent–child relationship		0.01
Preschool screen dependency behavior		
Effortful control	0.05	0.03
Mindful parenting	0.12	0.02
Parenting stress		0.39

All focal constructs in this study were specified as reflective constructs. In a reflective measurement model, the observed indicators are treated as manifestations of the same underlying latent variable rather than as formative components constituting the construct. Therefore, removing a limited number of low-loading or redundant indicators does not necessarily alter the substantive meaning of the construct, as long as the retained indicators continue to adequately reflect its core conceptual content (Bollen, 1989; Jarvis et al., 2003; Bollen and Lennox, 1991). Classical SEM literature also suggests that a latent construct should retain at least three observed indicators to support adequate model identification and representation (Bollen, 1989).

In the present study, each construct still retained more than three indicators after item trimming, and the main conceptual dimensions of the original scales remained adequately represented. Specifically, the CPRS used to measure parent–child relationship included the dimensions of closeness and conflict. After deleting CPRS2 and CPRS5, the closeness dimension retained five items; after deleting CPRS15, the conflict dimension retained seven items. The MIPQ used to measure mindful parenting included two dimensions. After deleting MIPQ1, MIPQ4, and MIPQ5, the “Being in the Moment with the Child” dimension retained 11 items; after deleting MIPQ15, the “Mindful Discipline” dimension retained 13 items. Parenting stress, which served as a control variable rather than a focal theoretical construct, retained six of its seven indicators after deleting PSS1. Thus, the retained indicators still covered the major conceptual content of the original measures, and the revised scales should be understood as shortened reflective measures rather than substantively redefined constructs.

The results of subsequent reliability and validity tests further supported the adequacy of the revised measurement model. The composite reliability (CR) and Cronbach’s alpha values for all latent variables exceeded 0.70, and all AVE values were above 0.50, meeting the recommended thresholds (Hair et al., 2019). In addition, according to the Fornell–Larcker criterion (Fornell and Larcker, 1981), the square roots of AVE for each latent variable were higher than the correlations with other constructs, supporting discriminant validity. Similarly, all heterotrait-monotrait ratio of correlations (HTMT) values were below 0.90, meeting the threshold suggested by Henseler et al. (2015). As for the explanatory power index, the *R*^2^ of preschool screen dependency behavior was 0.69, and the *R*^2^ of parent–child relationship was 0.49, indicating that the independent variables explained 69 and 49% of their variance, respectively. The effect sizes (*f*^2^) ranged from 0.01 to 0.12 (i.e., MIPQ → CPSR = 0.12; MIPQ → CPSD = 0.02; CPSR → CPSD = 0.01), which were acceptable according to the standards of Cohen (2013). Overall, the above results indicate that the revised measurement model has good reliability, validity, and explanatory power, and can proceed to the hypothesis testing stage.

### 4.2 Direct effect analysis

**Figure 2:**
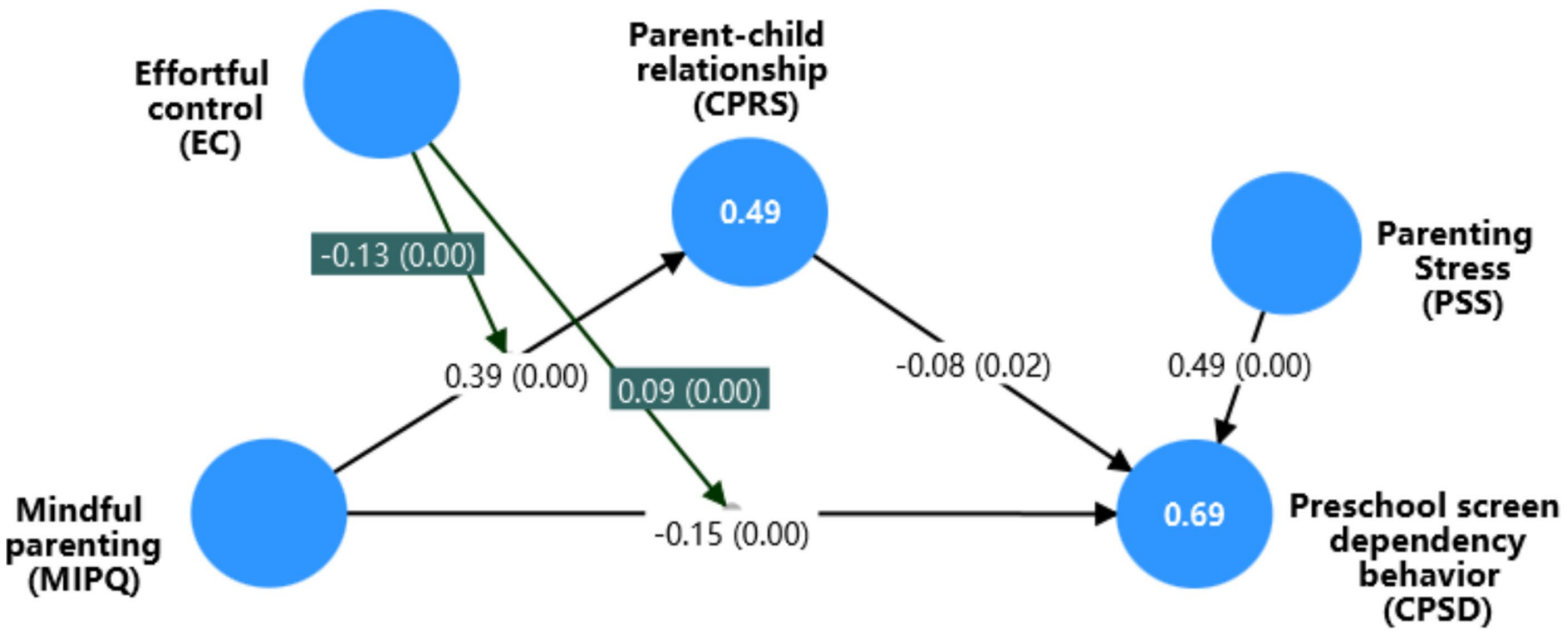
A structural model with moderated mediation.

During hypothesis testing, the bootstrap method with 5,000 resamples was applied to assess the significance of path coefficients. The analysis output includes path coefficients (*β*), *t*-values, confidence intervals (CI), and *p*-values, which are used to determine the significance and direction of each hypothesis. Table 2 shows that mindful parenting is significantly negatively associated with screen dependency behavior in preschoolers (*β* = −0.15, *t* = 3.28, *p* < 0.01), suggesting a negative correlation between high levels of mindful parenting and lower levels of screen dependency behavior in preschool children. Meanwhile, mindful parenting is significantly positively associated with parent–child relationship (*β* = 0.39, *t* = 7.51, p < 0.01), suggesting a positive correlation between higher levels of mindful parenting and a better parent–child relationship. Parent–child relationship is significantly negatively associated with screen dependency behavior in preschoolers (*β* = −0.08, *t* = 2.28, *p* < 0.05), indicating that higher-quality parent–child relationship is associated with lower levels of children’s screen dependency behavior. Thus, the results support H1–H3. The results of PLS-SEM analysis of the proposed model are shown in Figure 2.

**Table 2:** Hypothesis results.

Hypothesis	Paths	*β*	SD	*t* value	*p* value	2.5%	97.5%
H1	MIPQ → CPSD	−0.15	0.05	3.28	0.00	−0.24	−0.06
H2	MIPQ → CPRS	0.39	0.05	7.51	0.00	0.30	0.50
H3	CPRS → CPSD	−0.08	0.03	2.28	0.02	−0.14	−0.01
H4	MIPQ → CPRS → CPSD	−0.03	0.01	2.14	0.03	−0.06	−0.01
H5	EC × MIPQ → CPRS	−0.13	0.04	3.54	0.00	−0.19	−0.05
H6	EC × MIPQ → CPSD	0.09	0.03	3.02	0.00	0.03	0.15
H7	EC × MIPQ → CPRS → CPSD	0.01	0.01	1.81	0.07	0.00	0.02

MIPQ, mindful parenting; CPSD, preschool screen dependency behavior; CPRS, parent–child relationship; EC, effortful control.

### 4.3 Mediation analysis

After testing the direct effect, this study further examined the mediation hypothesis. The results indicate that parent–child relationship acts as a key mediator in the correlation between mindful parenting and preschoolers’ screen dependency behavior (*β* = −0.03, *t* = 2.14, *p* < 0.05). Given that zero is not included in the confidence interval, parent–child relationship mediates the link between mindful parenting and screen dependency behavior. In other words, mindful parenting is not only directly associated with preschoolers’ screen dependency behavior, but also indirectly associated with it through its positive association with parent–child relationship. Thus, H4 is supported.

### 4.4 Moderation analysis

**Figure 3:**
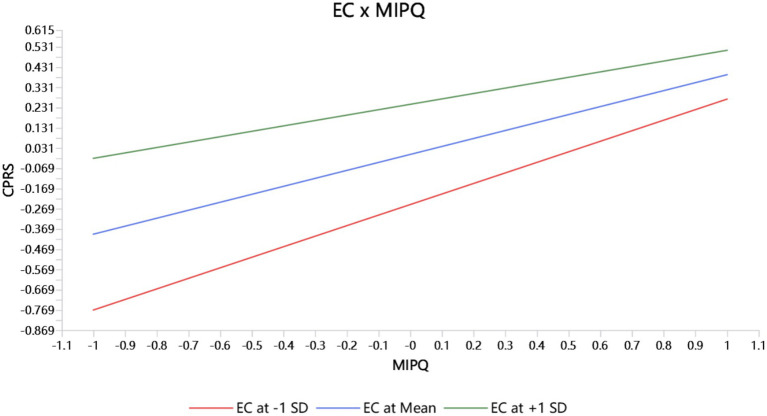
Moderation on parent–child relationship (CPRS).

**Figure 4:**
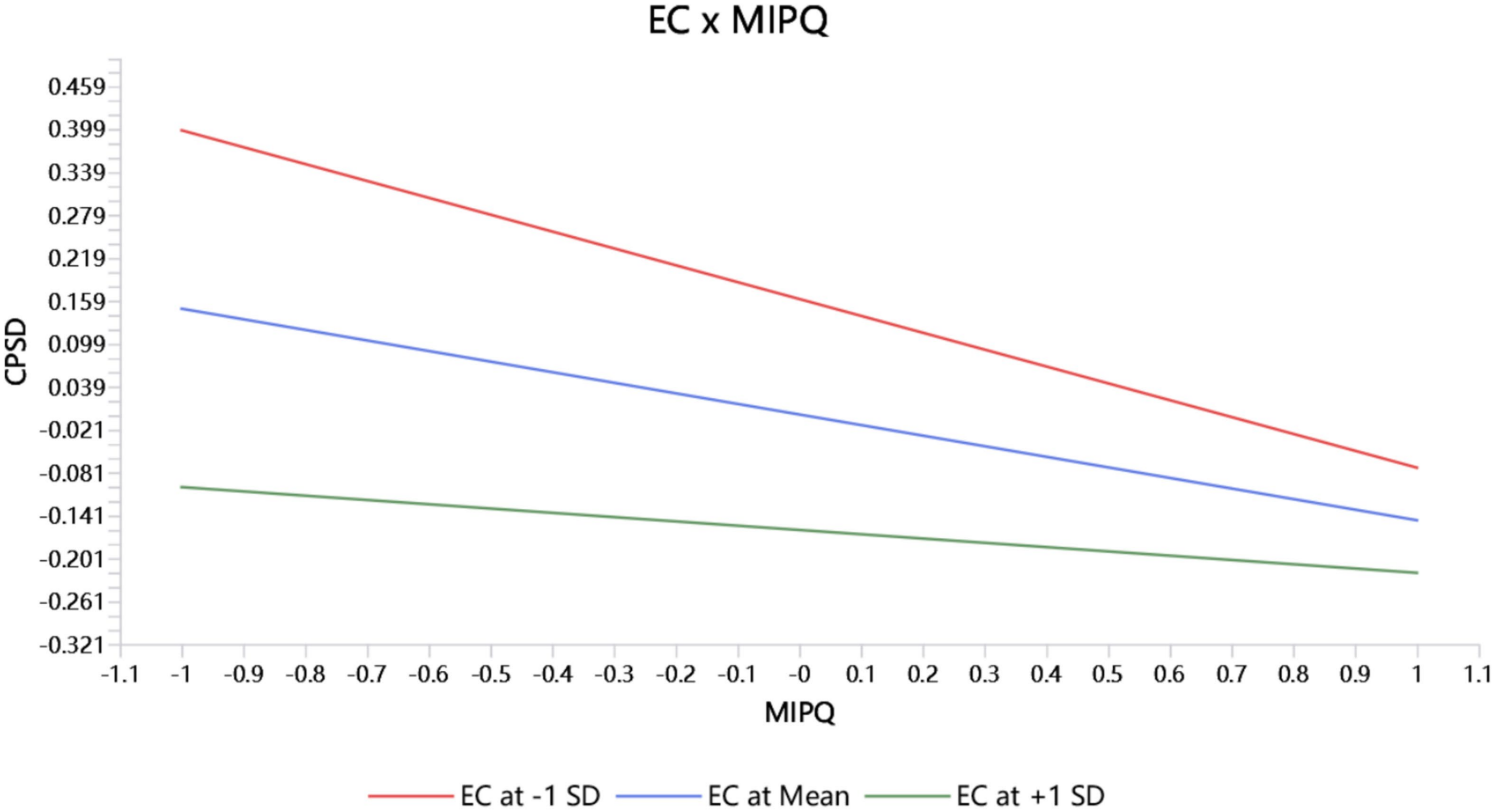
Moderation on screen dependency behavior (CPSD).

Subsequently, this study tested the moderation hypothesis. The results indicate that preschoolers’ effortful control has a significant negative moderating effect on the association between mindful parenting and parent–child relationship (*β* = −0.13, *t* = 3.54, *p* < 0.01). This indicates that for children with higher effortful control, the positive association between mindful parenting and parent–child relationship is weaker. Conversely, for children with lower effortful control, the association is more pronounced. Thus, H5 is supported (see Figure 3). Furthermore, effortful control plays a positive moderating role in the association between mindful parenting and screen dependency behavior (*β* = 0.09, *t* = 3.02, *p* < 0.01). Specifically, the negative association between mindful parenting and screen dependency behavior is weaker for children with higher effortful control, but stronger for those with lower effortful control. H6 is supported (see Figure 4). However, the moderating effect of effortful control on the mediation pathway is not significant [*β* = 0.01, *t* = 1.81, *p* > 0.05, 95% CI (0.00, 0.02)], though the confidence interval suggests a possible small effect. Thus, H7 is not supported. This implies that effortful control may not be significantly associated with the mediating role of parent–child relationship.

## 5 Discussion

This study investigated the association of mindful parenting with screen dependency behavior in preschoolers, together with the mediating role of parent–child relationship and the moderating role of effortful control. The results extend the application of mindful parenting theory to digital media contexts, and provide relevant support for family systems theory and the temperament-environment interaction model.

### 5.1 The direct association between mindful parenting and screen dependency behavior

A negative correlation emerged between mindful parenting and screen dependency behavior in this study, which aligns with prior empirical work suggesting that mindful parenting correlates with fewer behavioral problems in children (Stockdale et al., 2018; Vernon and Moretti, 2024). Mindful parenting, as a positive parenting style, shows a significant positive correlation with children’s prosocial behaviors, reflecting that such parenting corresponds to higher levels of positive behaviors (Cheung et al., 2021). Furthermore, mindful parenting correlates with fewer internalizing and externalizing problems in children (Vernon and Moretti, 2024). In the current digital era, parents who report lower levels of mindful parenting tend to permit extended video watching by their children during crying episodes, often seeking a short period of calm. This tendency may be related to an unintended strengthening of the emotional connection between children and screens, which in turn may be associated with more frequent screen use. Therefore, mindful parenting, as a conscious parenting approach, may be related to lower levels of children’s screen dependency behavior in a complex media environment, and may be associated with more stable and sustainable development for children.

### 5.2 Parent–child relationship as a mediating mechanism

This study found that mindful parenting is associated with preschoolers’ screen dependency behavior, and this association may be related to parent–child relationship, which is consistent with attachment theory and family systems theory (Bowlby, 1982; Huynh et al., 2024). The level of mindful parenting is related to the establishment of stable and supportive parent–child relationships (He et al., 2024). Children with secure and supportive parent–child relationships perform better in social–emotional development and have lower rates of behavioral problems (Zhang et al., 2019). Mindful parenting is associated with the formation of strong parent–child relationships and the development of healthier screen usage habits in children (Zhang, 2025). Conversely, poor parent–child relationships are more likely to be associated with seeking psychological comfort through excessive screen use (Zhu et al., 2022). This study shows that higher mindful parenting correlates with a better parent–child relationship, and that better parent–child relationship correlates with lower screen dependency behavior in preschoolers. Accordingly, parent–child relationship appears as a correlate linking mindful parenting to children’s screen dependency behavior. This mediation pathway connects media-related risks to family harmony and healthy child development.

### 5.3 Effortful control as a moderating mechanism

This study identified effortful control as a moderator in the link between mindful parenting and children’s screen dependency behavior. This finding is consistent with the temperament-environment interaction theory (Putnam and Rothbart, 2006). Effortful control, as a core component of temperament, is associated with children’s ability to regulate emotional and behavioral responses through attention and cognitive processes. Existing research has found that effortful control is associated with dual characteristics of protection and compensation (Gartstein and Fagot, 2003). This means that it may be related to buffering the negative effects of external stressors, fulfilling its protective function. It may also, to some extent, weaken the association with external interventions, reflecting its compensatory function.

This study found that effortful control is associated with a negative moderating role between mindful parenting and parent–child relationship. For children with low effortful control, this association is more pronounced. This is consistent with the differential susceptibility theory (Belsky, 1997). Individuals with vulnerability traits are more sensitive to environmental inputs. At the same time, this also reflects the protective function of effortful control for individuals. When children’s effortful control abilities are insufficient, mindful parenting may be associated with compensating for this deficiency and with protection from adverse environmental influences. Conversely, children with high effortful control have a stronger ability to self-regulate their emotional and behavioral responses, which is associated with lower dependence on external relational support. A good parent–child relationship can be maintained even when parents do not use mindful parenting strategies, and this relationship is linked to a smaller marginal effect of mindful parenting. This reflects the compensatory function of effortful control for individuals, meaning that high effortful control may be associated with partially compensating for the lack of external parenting support.

According to this study, effortful control positively moderates the direct relationship between mindful parenting and preschool children’s screen dependency behavior. For children with low effortful control, this association is more pronounced. This result confirms the protective function of effortful control. When children’s effortful control abilities are insufficient, mindful parenting may be associated with compensating for this deficit and with lower levels of screen dependence. In contrast, for children with high levels of effortful control, this association is weaker. This is because children with high levels of effortful control possess strong self-regulation abilities, allowing them to manage screen time independently, and this in turn is associated with less reliance on parenting styles. In this case, the additional space for the association with mindful parenting is smaller. This reflects the compensatory function of effortful control, meaning that high effortful control is associated with autonomous regulation of media use behavior, partially corresponding to the effects of parenting interventions. These findings are consistent with Coe’s view (Coe et al., 2024) that children with high levels of effortful control are less influenced by external factors. When faced with the family media environment or other pressures, their behavior is relatively stable. Effortful control may be associated with protecting children from the negative impacts of adverse environments.

Notably, effortful control did not moderate the indirect association between mindful parenting and screen dependency behavior through parent–child relationship, indicating that this mediating pathway remained stable across different levels of effortful control. This finding is consistent with the path specificity and mechanism heterogeneity of the temperament-environment interaction model (Putnam and Rothbart, 2006). Effortful control selectively moderated the direct path of mindful parenting on preschoolers’ screen dependency behavior, but did not affect the mediated path through parent–child relationship. This difference arises because the direct path is more associated with children’s immediate behavioral regulation abilities, making it more susceptible to temperament levels. The core of the mediating pathway is the long-term socialization function of the parent–child relationship, which exhibits cross-temperament stability. This finding expands the application boundaries of the temperament-environment interaction theory.

### 5.4 Theoretical implications

First, based on the background of the digital era, this study highlights the significance of mindful parenting for children’s growth and development. While previous research has primarily focused on traditional parenting styles (e.g., authoritative and neglectful parenting) and their associations with children’s social–emotional development and academic performance, the present study shifts attention to mindful parenting and its practical value in alleviating preschoolers’ screen dependency behavior. This responds directly to the call for exploring adaptive parenting strategies in the digital age (Emiroğlu İlvan and Ceylan, 2023). By extending family education research from traditional domains to digital behavior governance, this study contributes to building a positive parenting framework within the digital ecosystem.

Second, the mediating role of parent–child relationship between mindful parenting and screen dependency behavior in preschoolers is demonstrated. This study extends the theoretical framework linking parenting strategies with children’s behavioral development, while also expanding the application of attachment theory in the digital context and providing important practical evidence for its contemporary evolution.

Third, a moderating role of children’s effortful control was identified in this study. The level of effortful control may be associated with children’s responses to the family environment (Rothbart et al., 2000). Children with high effortful control are less influenced by the environment, while children with low effortful control are more dependent on family support and guidance (Kochanska and Kim, 2014; Coe et al., 2024). This study deepens our understanding of the causes underlying individual differences in the relationship between family nurturing environments and early childhood developmental outcomes. The finding adds new empirical evidence to developmental psychology research concerning the interaction between children’s self-regulation ability and their environment.

### 5.5 Practical implications

First, parents’ mindful parenting skills should be enhanced. With the widespread use of screen media in family life, children’s screen exposure opportunities and frequency have increased significantly. Parents need to constantly improve their mindful parenting skills, as this may be associated with lower levels of screen dependency behaviors in children. Parents can enhance their present-moment awareness, emotional regulation and non-judgmental responses through structured mindfulness training, family education seminars, or community parent–child interaction workshops. In addition, the principles of mindfulness can be incorporated into the guidelines for daily interactions and the use of family media. Parents should also take the initiative to understand the development trends and potential risks of digital media. When discussing an appropriate screen schedule with children, they should maintain open communication and provide a clear behavioral framework, so as to guide children to use screen media more effectively in the digital environment.

Second, a supportive parent–child interaction system should be established. Families should focus on creating a stable and warm supportive parent–child interaction pattern. Through diverse activities such as shared reading and cooperative games, they can cultivate a secure attachment relationship in daily interactions, effectively enhancing the quality of parent–child relationship. This high-quality interaction can create a secure psychological environment for children. It may also lay an emotional foundation for subsequent skill development and behavioral guidance, enabling children to form positive self-awareness and emotional connection in a relationship full of acceptance and response.

Third, families and kindergartens should collaborate to cultivate children’s effortful control. Effortful control is associated with children’s ability to manage screen dependency behavior. Given the high plasticity of children, families and kindergartens can systematically cultivate their effortful control through life-oriented and game-oriented methods. This may be associated with building a foundation for managing screen dependency and other behavioral problems in the future. In daily family life, parents can create some control practice situations and let children experience delayed gratification in real choices through rule games, such as waiting for a while before eating snacks. When children show self-control behaviors, parents should give timely affirmation to strengthen children’s positive experience. Kindergarten teachers can integrate effortful control training into daily activities, such as taking turns in games, listening quietly, and completing tasks step by step, so that children can practice suppressing impulses and sticking to goals in a group setting. Teachers help children understand the strategy of “stop, think, and then act” through demonstration and guidance. When conflicts arise, they support children’s behavior regulation by combining empathy with clear rules.

### 5.6 Research limitations and future prospects

Several limitations of the present study warrant attention. First, the factors associated with screen dependency behavior in preschoolers were explored using a cross-sectional design. However, given constraints of time and resources, longitudinal tracking was not carried out, which limits the ability to infer causal relationships among the variables. The mediation hypothesis was tested as a theoretically derived indirect association rather than a temporal causal chain. This cross-sectional analysis therefore examined the indirect pathway as a theoretical implication of family systems theory and attachment theory, without claiming strict causal ordering. Therefore, longitudinal design or experimental intervention methods can be adopted in future research to reveal the influence mechanism of mindful parenting on preschoolers’ screen dependency behavior more accurately.

Second, the parent–child relationship and the effortful control of preschoolers are mainly investigated as mediating and moderating variables in this study, while other potential variables, such as parents’ screen use behaviors and family media use rules, are not included. Future research may further explore the interaction between the family digital environment and early childhood characteristics, formulate more targeted and layered intervention programs, and achieve precise support and sustainable behavioral improvement.

Third, the sample selected in this study was mainly drawn from urban kindergartens, and the survey participants were mainly mothers. The representativeness of the sample is limited. In addition, the data were mainly based on parents’ self-reports, which is prone to subjective bias. Additionally, the unsupervised home completion of questionnaires may introduce uncontrolled variability, such as external distractions or third-party influence, which was not directly assessed in this study. These factors may have implications for the stability and generalizability of the conclusions. In future research, the sample coverage could be expanded to include rural and diverse regional family samples, thereby enhancing the generalizability of the findings. During data collection, it would be essential to integrate multiple sources of information, including parents’ and teachers’ assessments, and direct observations of children’s behaviors. Researchers can adopt a triangulation method to reduce biases in subjective reports, thus improving the reliability and objectivity of the research conclusions.

## 6 Conclusion

This study investigated the link between mindful parenting and screen dependency behavior in preschoolers, together with the mediating role of parent–child relationship and the moderating effect of effortful control. For children with lower effortful control, the correlation between mindful parenting and improvements in parent–child relationship, as well as reductions in screen dependency behavior, is significantly more pronounced. However, the mediating pathways of parent–child relationship show stability across different levels of effortful control. This differentiated pattern of “sensitive direct pathways and robust mediating pathways” is consistent with the principle of path specificity in temperament-environment interactions. In practice, this means that direct behavioral interventions related to mindful parenting need to be adapted to the children’s effortful control levels, while parent–child relationship interventions may be applicable to a broader range of children. This study provides practical support for the combination of individualized instruction and universal support in early childhood education.

## Data Availability

The original contributions presented in the study are included in the article/Supplementary material, further inquiries can be directed to the corresponding author.
